# Acetate suppresses myocardial contraction *via* the short-chain fatty acid receptor GPR43

**DOI:** 10.3389/fphys.2022.1111156

**Published:** 2022-12-16

**Authors:** Xuan Jiang, Ying Zhang, Huaxing Zhang, Xiaoguang Zhang, Xiaopeng Yin, Fang Yuan, Sheng Wang, Yanming Tian

**Affiliations:** ^1^ Department of Neurobiology, Hebei Medical University, Shijiazhuang, Hebei, China; ^2^ Department of Physiology, Hebei Medical University, Shijiazhuang, Hebei, China; ^3^ Core Facilities and Centers, Hebei Medical University, Shijiazhuang, China; ^4^ Hebei Key Laboratory of Neurophysiology, Shijiazhuang, Hebei, China

**Keywords:** acetate, short-chain fatty acids, cardiac contractility, calcium handling, GPR43, FFAR2

## Abstract

The heart has high energy requirements, with an estimated 40%–60% of myocardial ATP production derived from the oxidation of fatty acids under physiological conditions. However, the effect of short-chain fatty acids on myocardial contraction remains controversial, warranting further research. The present study sought to investigate the effects and mechanisms of acetate, a short-chain fatty acid, on myocardial contraction in rat ventricular myocytes. Echocardiography and Langendorff heart perfusion were used to evaluate cardiac function. Cell shortening and calcium transient were measured in isolated cardiomyocytes. The patch-clamp method determined the action potential and L-type Ca^2+^ current in cardiomyocytes. Moreover, the expression of GPR43, a type of short-chain fatty acid receptors in cardiomyocytes was examined by immunofluorescent staining and Western blot. We demonstrated that acetate transiently reduced left ventricular developmental pressure in isolated Langendorff heart perfusion model, with no effect on stroke volume and cardiac output *in vivo*. In addition, acetate transiently and reversibly inhibited cardiomyocyte contraction and calcium transient. Acetate did not affect the action potential and L-type Ca^2+^ currents in cardiomyocytes. As a short-chain fatty acid receptor, GPR43 was expressed in rat cardiomyocytes. Furthermore, the GPR43 antagonist GLPG0974 prevented the acetate-induced inhibitory effect on myocardial contraction. We conclude that acetate transiently inhibits contraction *via* the short-chain fatty acid receptor GPR43 in cardiomyocytes.

## 1 Introduction

It is well-established that the heart has high energy requirements, given that the myocardium must contract and relax continuously to pump blood. Accordingly, the heart generates large amounts of ATP to sustain contractile function (Kolwicz et al., 2013), achieved by utilizing a variety of energy substrates, including fatty acids, glucose, lactate, ketones, pyruvate, and amino acids (Schulze and Wu, 2020). Current evidence suggests that 40%–60% of myocardial ATP production are derived from the oxidation of fatty acids under physiological conditions (Lopaschuk et al., 2010). Fatty acids are divided into long-chain fatty acids (LCFAs, >12 carbons), medium-chain fatty acids (MCFAs, 6–12 carbons) and short-chain fatty acids (SCFAs, <6 carbons) according to the length of the carbon chain. Recent studies have shown that the supplementation of palmitic acid (an LCFA) could enhance contraction tension in cardiomyocyte (Jeong et al., 2018). However, the role of SCFA in myocardial contraction remains controversial.

SCFAs, including acetate, propionate and butyrate, are predominantly produced by fermentation of indigestible dietary fiber in the intestine by the gut bacteria, and play a crucial role in cardiac energy metabolism in health and disease (Hu et al., 2022). In addition to being involved in energy metabolism as substrates, SCFAs can act as signaling molecules to activate G protein-coupled receptors (GPCR), such as GPR41, GPR43 and GPR109A, followed by activation of intracellular calcium or cyclic adenosine monophosphate (cAMP) *via* G protein (Gq or Gi/o)-dependent pathways, and regulate intracellular signal transduction (Lymperopoulos et al., 2022). As the most abundant SCFA, acetate has long been studied on myocardial energy metabolism and contraction function (Hu et al., 2018), but studies have yielded inconsistent results, with reports of enhanced, inhibited and unaltered myocardial contraction after acetate treatment (Kirkendol et al., 1978; Liang and Lowenstein, 1978; Jacob et al., 1997; Martin et al., 1998).

In the present study, we evaluated the effect of acetate on myocardial contraction at the global heart and isolated cellular levels in adult rats. Cardiomyocyte contraction was assessed after treatment with GPR43 antagonist GLPG0974.

## 2 Materials and methods

### 2.1 Animals

Adult male Wistar rats (12–14 weeks old, body weight 250–300 g) were purchased from Beijing Vital River Laboratory Animal Technology Co., Ltd. The rats were housed in the IVC system (22°C–24°C, relative humidity 50%–60%), with a 12 h/12 h light/dark cycle, and *ad libitum* access to food and water. All experiments were conducted in accordance with the Guide for the Care and Use of Laboratory Animals, and were approved by the Animal Care and Ethics Committee of Hebei Medical University.

### 2.2 Echocardiography

Rats were anesthetized with isoflurane (induced with 2.5%, maintained with 1.5%), and secured to the operating table in a supine position. After shaving the chest hairs and venous cannula procedure, the rats were examined by left ventricular long axis B-mode ultrasound and left ventricular outflow tract pulsatile wave (PW) Doppler, with a Vevo 2100 Imaging Systerm (Fujifilm VisualSonics, Canada) with a high-frequency linear array transducer probe (MS250, Fujifilm VisualSonics). Sodium acetate (67.5 mM, 0.1 ml, pH 7.4, Sigma-Aldrich, United States) was administered intravenously. The concentration of sodium acetate in this *in vivo* experiment was referred to previous studies (Erny et al., 2015; van de Wouw et al., 2018). The obtained left ventricle outflow tract diameter (D), velocity-time integral (VTI) and cardiac cycle were used to calculate the heart rate (HR), stroke volume (SV) and cardiac output (CO) according to the following formulas: SV = *π* × (D/2)^2^ ×VTI, CO = SV × HR. Each data set was obtained from the average of three consecutive cardiac cycles.

### 2.3 Cardiac function recording *in vitro*

Rats were anesthetized with urethane (1.25 g/kg, i. p.), and the hearts were excised and fixed on a Langendorff apparatus, and retrogradely perfused with Krebs-Henseleit (K-H) solution at stationary pressure (10 kPa) *via* the aorta. The components of K-H solution included (in mM): NaCl 118, NaHCO_3_ 25, glucose 11, KCl 4.7, CaCl_2_ 2.5, MgSO_4_ 1.2, and KH_2_PO_4_ 1.2 (steadily bubbled with 95% O_2_ and 5% CO_2_, 37°C, pH 7.4). The latex balloon-tipped catheter filled with deionized water was inserted into the left ventricle through the left atrium and the left ventricular end-diastolic pressure (LVEDP) was adjusted to ∼5 mmHg during the stable period (20 min). After the equilibration period, if the LVEDP below 0 mmHg, the LVEDP would be readjusted and continue to be stable. If the LVEDP exceeded 10 mmHg after the equilibration period, the heart and data will be discarded. The distal end of the catheter was connected to a pressure transducer (AD Instrument, Australia), and the pressure signal was recorded with the PowerLab system (AD Instrument). Left ventricular developed pressure (LVDP), the maximal left ventricular developed pressure (LVDP_max_), the maximal differentials of LVDP (±dp/dt_max_), LVEDP and heart rate were continuously recorded. After 20 min of stabilization, the hearts were perfusion with sodium acetate (1 mM, pH 7.4) mixed with K-H solution for 5 min. The concentration of sodium acetate was referred to a previous study (Poll et al., 2021), and modified according to our preliminary experiments. The data were analyzed by LabChart 7 software (AD Instrument).

### 2.4 Myocytes isolation and cell shortening measurement

The heart was taken out and fixed on a Langendorff apparatus after the rat was anesthetized and perfused with Ca^2+^-free Tyrode’s solution containing type II collagenase (0.4 g/L, Roche, Switzerland) through the aorta for 20–30 min. The ventricles were cut into pieces, filtered with a strainer (200 meshes), and then incubated with fresh KB solution. The KB solution (pH 7.2) contained (mM): Na-β-OH-Butyric Acid 5, creatine 5, Taurine 20, EGTA 0.5, NaOH(EGTA) 1.09, MgSO_4_·7H_2_O 5, HEPES 5, Glucose 10, KCl 90, K_2_HPO_4_ 30, and Na-Pyruvic Acid 5. Tyrode’s solution (pH 7.4) consisted of (mM): NaCl 137.3, KCl 5.4, NaOH 2.7, MgCl_2_ 1, Glucose 10, and HEPES 5.

The single-cell contraction assay was conducted using the SoftEdge MyoCam system (Ion Optix Corporation, Milton, MA, United States). The isolated cardiomyocytes were allowed to stand for 2 h and incubated with Tyrode’s solution containing Ca^2+^ (1.8 mM) for 30 min. Cells were stimulated stably (5 m, 10 V, 1 Hz) for 2 min before sodium acetate (final concentration with 2 mM) or GLPG0974 (final concentration with 10 μM, Cat# SML2443, Sigma-Aldrich, United States) was given, and rod-shaped myocytes exhibiting clear striations were selected for experiments. The concentration of sodium acetate and GLPG0974 were referred to previous studies (Schooley et al., 2014; Li et al., 2018), and modified according to our preliminary experiments. The MyoCam system of IonOptix converted the signal, which was automatically acquired and recorded in real time. The results were processed by IonOptix SoftEdge software. The cell contractility was evaluated by the sarcomere contraction amplitude (ΔSL%). At the same time, maximum contraction velocity (dep v) and maximum relaxation velocity (ret v) were analyzed as contraction indexes.

### 2.5 Measuring calcium transient and calcium storage in the sarcoplasmic reticulum

Following Langendorff perfusion and enzymatic digestion, the cardiomyocytes were loaded with Fluo-4-acetoxymethyl ester (2 μM, Fluo-4 AM, Invitrogen, United States) and F127 (Thermo Fisher, United States) mixed 1:1 in Tyrode’s solution containing Ca^2+^ (1.8 mM) at room temperature. The supernatant liquid was replaced after 30 min to remove the excess fluorescent dye. Cells were transferred to a chamber on a laser scanning confocal microscope (FV 10, Olympus, Japan). The stimulation mode and cell selection used were the same as mentioned above. Recordings were started after 2 min of stabilization during sodium acetate (2 mM) stimulation. Simultaneously with the electrical stimulation (as above), the Fluo-4 fluorescence signal excited by the excitation light at 488 nm was recorded under 40-fold magnification. To observe the effect of acetate on calcium reserve of sarcoplasmic reticulum, a high concentration of caffeine (10 mM, Aladdin, China) was given to induce calcium release from the sarcoplasmic reticulum. ImageJ (NIH, United States) and Clampfit 10.4 (Molecular Devices Corporation, United States) software were used to analyze the calcium transient amplitude (F/F0) and calcium recovery rate (τ).

### 2.6 Measurement of L-type calcium current and action potential

The whole-cell patch-clamp method was applied to observe cell membrane current. Patch-pipette resistances were 3–5 MΩ before sealing. The pipette solution contained (in mM) CsCl 110, TEA-Cl 20, Glucose 10, HEPES 5, Mg-ATP 5, and EGTA 10 (pH adjusted to 7.2 with CsOH). Cells were incubated with Tyrode’s solution containing Ca^2+^ (1.8 mM). The signals were passed through Axon 200B amplifier and A/D conversion (Digidata 1550, Axon Instrument, United States). The holding potential was gradually changed from -50 mV to +60 mV. Currents were elicited by 500 ms depolarizing voltage steps of 10 mV nominal increment. The experiment was conducted at room temperature (20–25°C), and the external solution was restored to room temperature before the experiment. The action potentials of ventricular myocytes were recorded with a whole-cell current clamp. The initial voltage is maintained at −70 mV. A 5 ms, 1000–1600 pA current was injected to induce an action potential. L-type calcium current and action potential were tested 1 min and 5 min after administration of sodium acetate (2 mM).

### 2.7 Immunofluorescence staining

Isolated cardiomyocytes were fixed with 4% paraformaldehyde for 3–5 min, washed with 0.01 M PBS and permeabilized with 0.5% Triton X-100 (Sigma, United States). After blocking with 10% normal goat serum (Solarbio, Beijing, China), cells were incubated with GPR43 (dilution 1:100, Cat# AFR-032, RRID: AB_2756592, Alomone labs, Israel) and the type 2 ryanodine receptor (RyR2, dilution 1:100, Cat# ab2827, RRID: AB_2183052, Abcam, United States) antibodies for 12 h in 4°C. After the primary antibody was washed, the Cy3 AffiniPure Goat Anti-Rabbit IgG (H + L) (dilution 1:100, Cat# 111-65-003, RRID: AB_2338000, Jackson, United States) and Fluorescein (FITC) AffiniPure Donkey Anti-Mouse IgG (H + L) (dilution 1:100, Cat# 715-095-150, RRID: AB_2340792, Jackson, United States) secondary antibodies were incubated at normal temperature for 1 h. Cells were spread evenly on slides and treated DAPI with Fluoromount-G (SouthernBiotech, United States). The images were acquired using a laser-scanning confocal microscope (LSM 800, Carl Zeiss, Germany).

### 2.8 Western blot analysis

The rat ventricular myocytes were added into the lysis buffer to extract the protein, and the absorbance was measured with a microplate reader (Multiskan SkyHigh, Thermo Fisher, United States) at 562 nm to quantify the protein concentration using the BCA Protein Assay Kit (Cat# SW101-02, Seven Biotech, Beijing, China). 12% acrylamide gel was prepared and 30 μg of total protein was added to each lane for electrophoresis. The proteins separated by electrophoresis were transferred to polyvinylidene fluoride (PVDF) membranes after 1 h. The PVDF membranes were blocked with 5% BSA (BioFroxx, Germany) at room temperature for 1 h and incubated with anti-GPR43 monoclonal antibody (dilution 1:500, Cat# AFR-032, RRID: AB_2756592, Alomone labs, Israel) and GAPDH (dilution 1:3000, Cat# EM1101, RRID AB_2811078, HuaAn Biotechnology, China) for 12 h in 4°C. After washing the membranes, they were incubated with secondary antibodies HRP-conjugated Affinipure Goat Anti-Rabbit IgG(H + L) (dilution 1:2000, Cat# SA00001-2, RRID: AB_2722564, Proteintech, United States) for 1 h at room temperature. After washing the antibodies, the protein signals were developed in a luminometer (ChemiScope S6, CLINX, Shanghai, China), and processed by ImageJ software.

### 2.9 Statistical analysis

Statistical analysis was performed using GraphPad Prism version 8 (GraphPad Software Inc., United States). All data were expressed as mean ± SEM and were assessed by repeated measures one-way ANOVA with Bonferroni *post hoc* tests, or unpaired Student’s *t* test. A *p*-value <0.05 was statistically significant.

## 3 Results

### 3.1 Acetate has no effect on cardiac output in anesthetized rats

**FIGURE 1:**
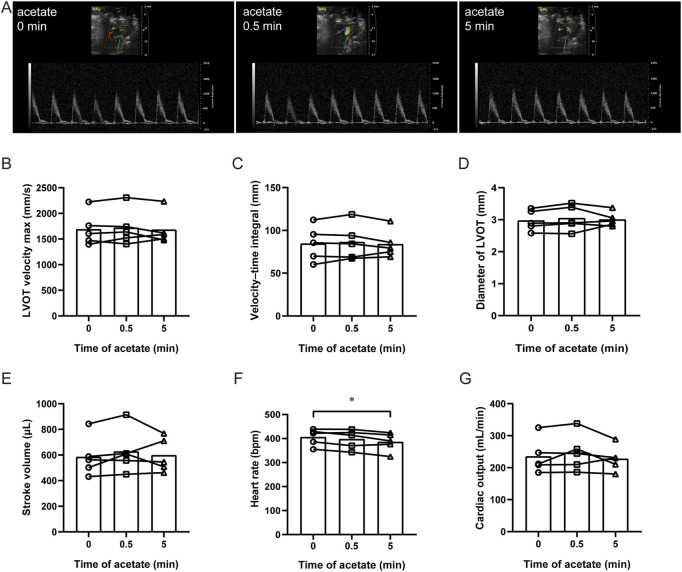
Acetate has no effect on cardiac output in rats. Representative original image of ultrasonic cardiogram in anesthetized rats by isoflurane **(A)**. The maximal blood flow velocity at the left ventricular outflow tract **(B)**, the velocity–time integral **(C)**, the diameter of the left ventricular outflow tract **(D)**, cardiac stroke volume **(E)**, heart rate **(F)**, and cardiac output **(G)** in anesthetized rats. *N* = 5. N: the number of rats. ^*^ *p* < 0.05.

We measured aortic hemodynamic parameters to assess cardiac pumping function using PW Doppler and analyzed its stroke volume, heart rate and cardiac output in anesthetized rats (Figure 1A). After acute administration of acetate, there were no significant changes in the maximal blood flow velocity at the left ventricular outflow tract (LVOT velocity max) (*p* > 0.05, Figure 1B), the velocity-time integral of LVOT (*p* > 0.05, Figure 1C), and the diameter of LVOT (*p* > 0.05, Figure 1D). Therefore there were no changes in stroke volume (*p* > 0.05, Figure 1E). Although, the heart rate decreased from 406 ± 16 to 386 ± 18 bpm 5 min after the injection (*p* < 0.05, Figure 1F), no significant difference was found in cardiac output (*p* > 0.05, Figure 1G). The same results were obtained with ultrasound-guided ventricular injection (data were not shown).

### 3.2 Acetate inhibits contractility in *ex vivo* heart

**FIGURE 2:**
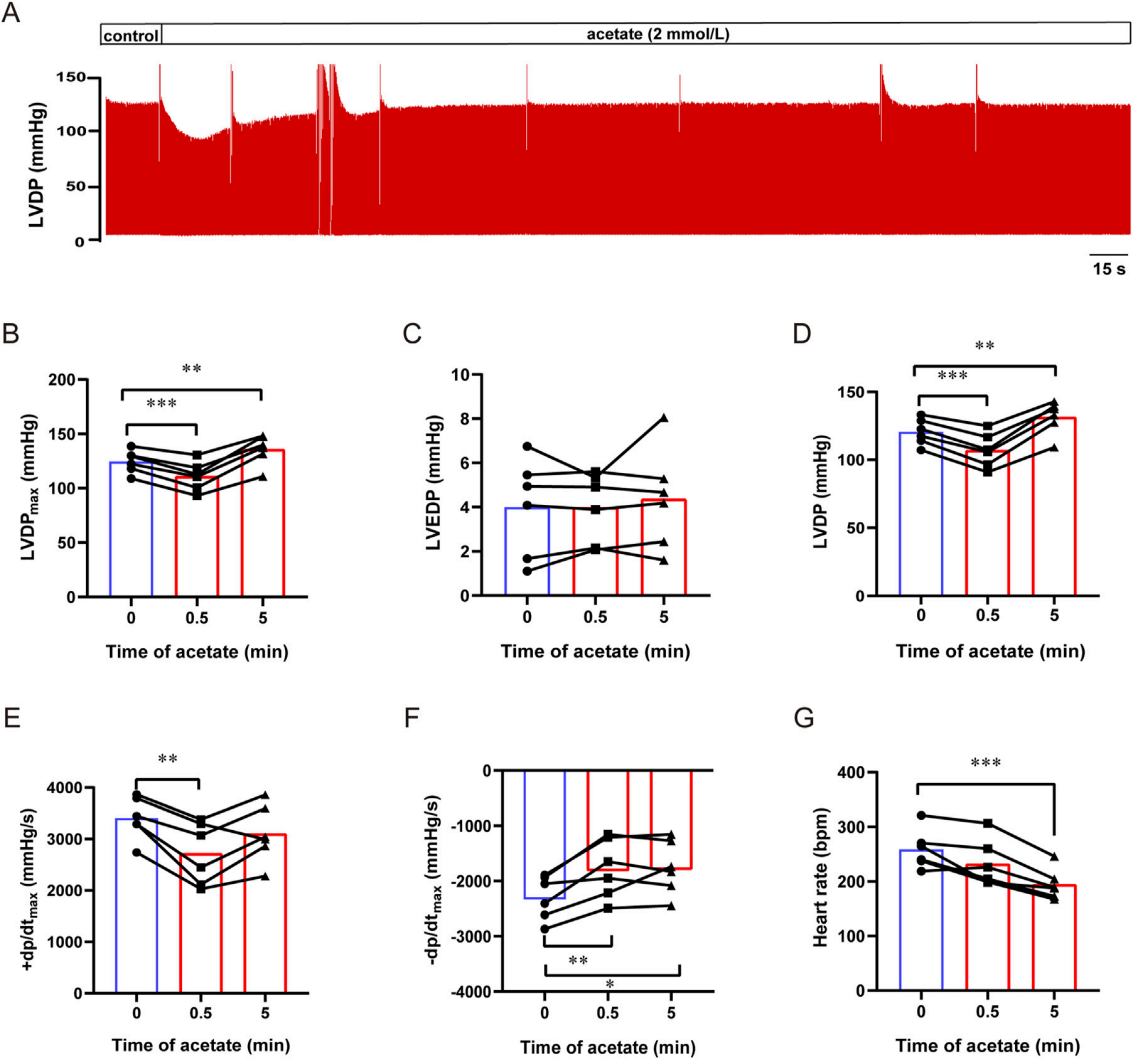
Acetate inhibited the contractility of Langendorff-perfused rat hearts. Representative traces of left ventricular pressure in isolated heart perfusion with acetate **(A)**. The maximal left ventricular developed pressure **(B)**, left ventricular end-diastolic pressure **(C)**, left ventricular developed pressure **(D)**, the maximal differentials of LVDP **(E,F)**, and heart rate **(G)** in Langendorff-perfused heart. *N* = 6. N: the number of rats. ^*^ *p* < 0.05, ^**^ *p* < 0.01, ^***^ *p* < 0.001.

To exclude the influence of neurological and humoral factors and observe the direct effect of acetate on the heart, we recorded the contractility of isolated hearts by langendorff perfusion (Figure 2A). After acetate perfusion, the LVDPmax transient decreased within 1 min (from 125 ± 4 to 111 ± 5 mmHg, *p* < 0.001, Figure 2B), and then recovered and slightly increased about 11.4 mmHg compared to baseline (*p* < 0.01, Figure 2B). Acetate perfusion had no effect on LVEDP (*p* > 0.05, Figure 2C), while there was a transient decrease and subsequent increase in LVDP (LVDPmax - LVEDP) after acetate infusion (Figure 2D). After acetate infusion, ±dp/dt showed a transient decrease (*p* < 0.01, Figures 2E,F), then + dp/dt, but not -dp/dt, returned to baseline. Moreover, acetate significantly reduced the heart rate of isolated hearts (from 259 ± 15 to 195 ± 12 bpm, *p* < 0.001, Figure 2G). These findings indicated that acetate could transiently inhibit the myocardial contractility *ex vivo*.

### 3.3 Acetate transiently inhibits the contraction of cardiomyocytes

**FIGURE 3:**
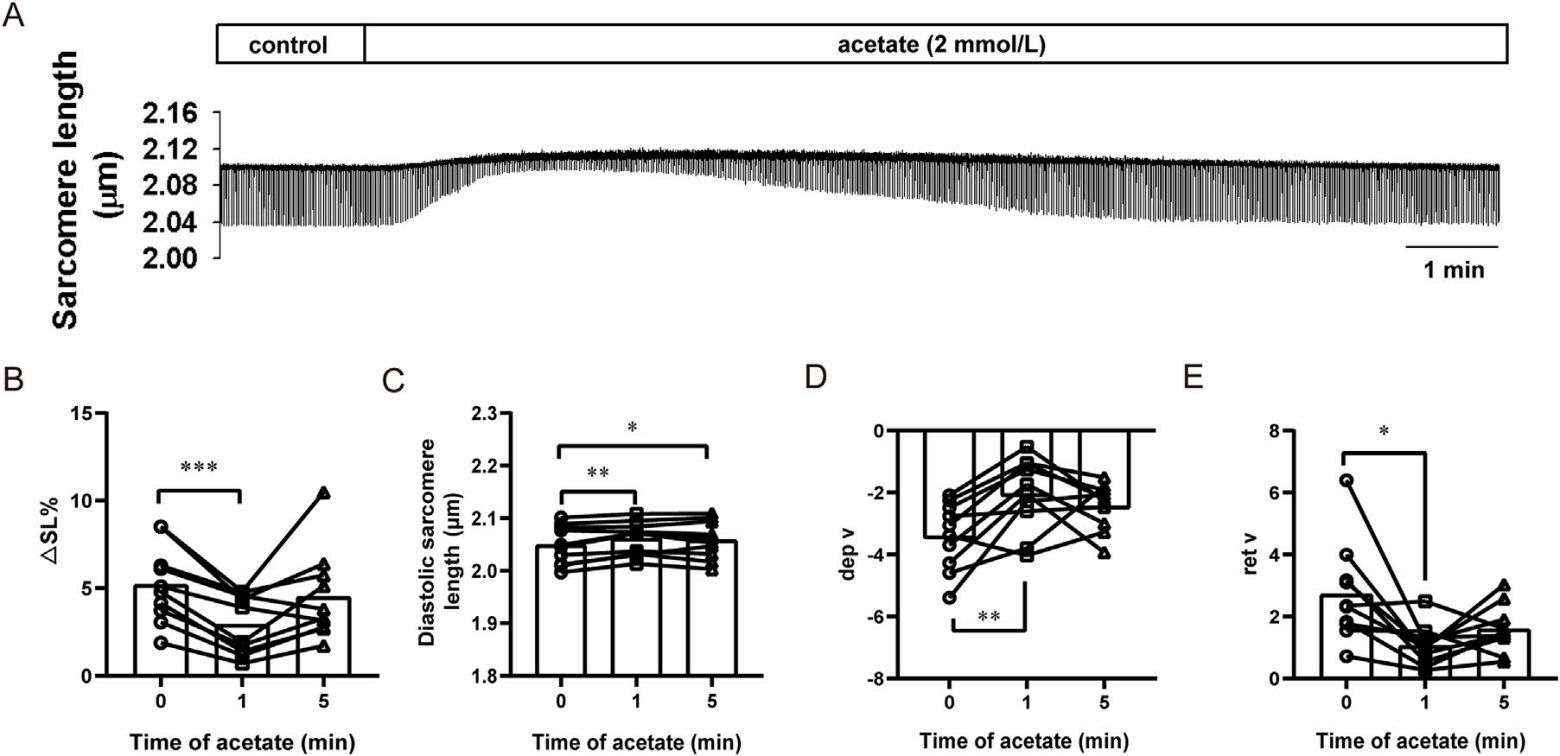
Acetate inhibited contractility in isolated myocardial cells. Representative traces of myocardial contraction **(A)**. The sarcomere contraction amplitude **(B)**, diastolic sarcomere length **(C)**, maximum contraction velocity **(D)**, and maximum relaxation velocity **(E)** in isolated myocardial cells. *N* = 5, *n* = 10. N: the number of rats, n: the number of cells. ^*^ *p* < 0.05, ^**^ *p* < 0.01, ^***^ *p* < 0.001.

We further observed the effect of acetate on the contraction of isolated cardiomyocytes (Figure 3A). We found that after incubation with 2 mM sodium acetate, the amplitude of contraction exhibited a transient decrease within 1 min (from 5.21 ± 0.69 to 2.93 ± 0.53, *p* < 0.001, Figure 3B) accompanied by an increased diastolic sarcomere length (from 2.05 ± 0.01 to 2.06 ± 0.01, *p* < 0.01, Figure 3C). Besides, decreased contraction velocity (from -3.40 ± 0.34 to -2.04 ± 0.37, *p* < 0.01, Figure 3D) and relaxation velocity (from 2.72 ± 0.50 to 1.06 ± 0.21, *p* < 0.05, Figure 3E) was found.

### 3.4 Acetate decreased the calcium transient amplitude

**FIGURE 4:**
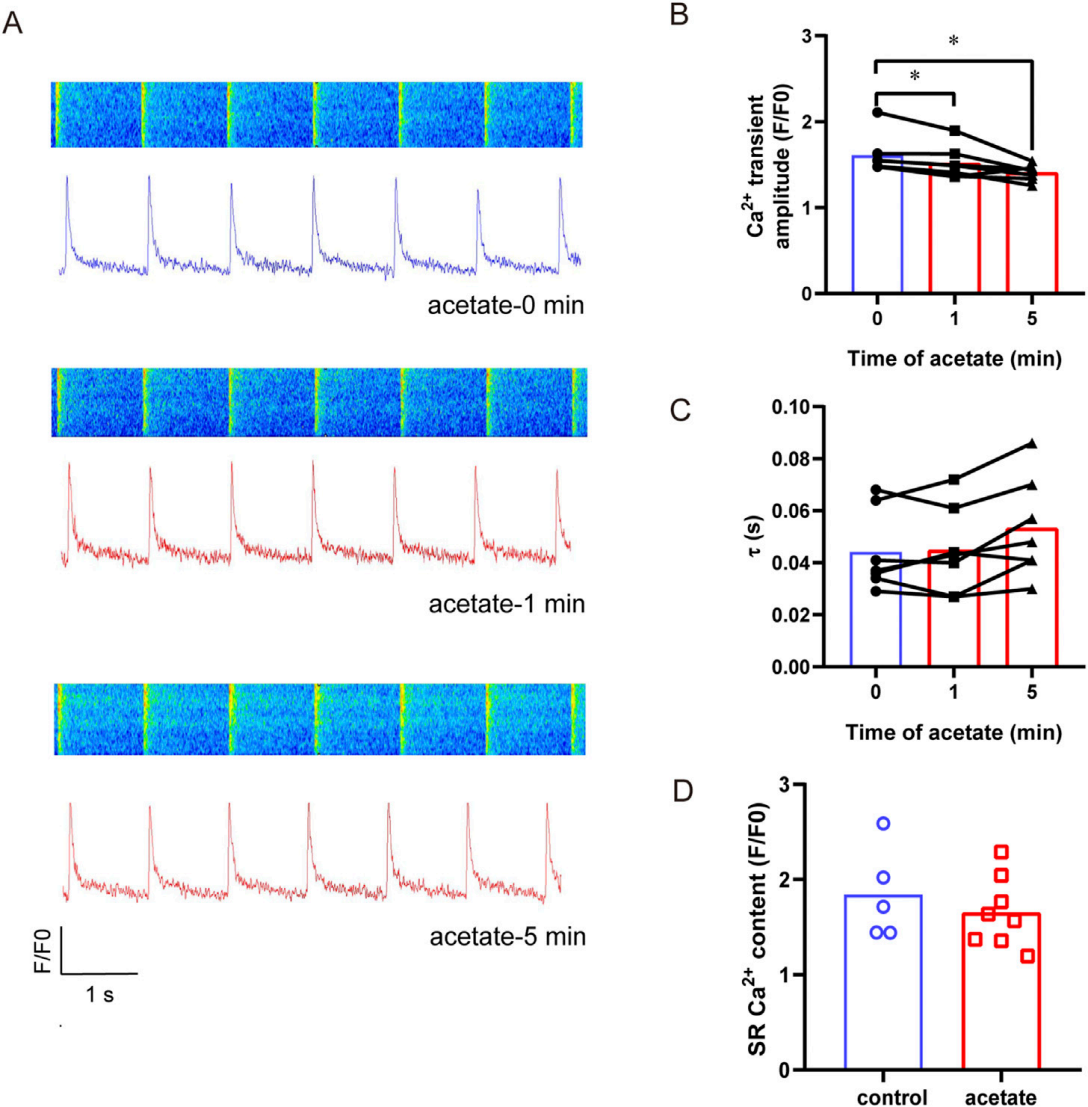
Acetate decreased the amplitude of calcium transient in rat cardiomyocytes. Original plot of calcium transient in isolated myocardial cell **(A)**. The calcium transient amplitude **(B)** and calcium recovery rate **(C)**, *N* = 5, *n* = 7. The Ca^2+^ content of sarcoplasmic reticulum in cardiomyocytes **(D)**, *N* = 5 for each group, *n* = 5 for control group, *n* = 8 for acetate group. N: the number of rats, n: the number of cells. ^*^ *p* < 0.05.

To reveal the mechanism underlying contraction inhibition induced by acetate, we directly measured Ca^2+^ concentration and sarcoplasmic reticulum calcium content in acetate-treated cardiomyocytes (Figure 4A). The calcium transient amplitude in the cardiomyocytes decreased significantly in 1 min after acetate administration (*p* < 0.05, Figure 4B), confirming a decrease in the concentration of cytoplasmic free calcium ions. However, the recovery rate (Figure 4C) and sarcoplasmic reticulum calcium content (Figure 4D) were not significantly changed (*p* > 0.05). These results showed that reduced free calcium ion concentration caused by acetate treatment.

### 3.5 Acetate failed to alter the action potential and L-type calcium channel current in cardiomyocytes

**FIGURE 5:**
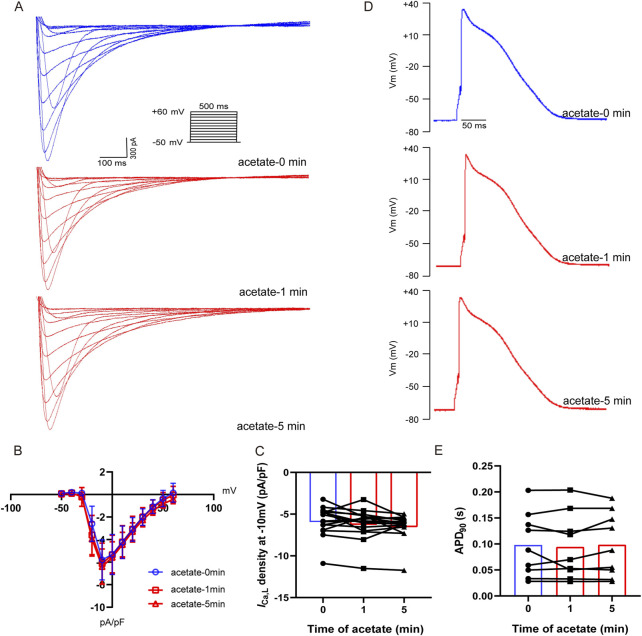
Acetate had no effect on the activity of L-type calcium current and action potential in rat ventricular myocytes. Recording of L-type calcium current from isolated cardiomyocytes **(A)**. Current density-voltage curve for L-type calcium current in cardiomyocytes with or without acetate **(B)**, *N* = 5, *n* = 16. The max current density for L-type calcium current in cardiomyocytes **(C)**, *N* = 5, *n* = 16. Representative action potential recordings in rat ventricular myocyte before and after perfusion with acetate **(D)**. 90% of the duration of action potential in cardiomyocytes **(E)**, *N* = 5, *n* = 9. N: the number of rats, n: the number of cells.

To investigate whether the depressed intracellular calcium concentration by acetate was related to decreased extracellular calcium influx, we recorded the *I*_Ca,L_ of ventricular myocytes after incubation with acetate. The *I-V* curve showed that acetate did not alter the activity of the L-type calcium current (Figures 5A, B). Current density (pA/pF) at -10 mV was not change significantly compared with the control ventricular myocytes (*p* > 0.05, Figure 5C). Similarly, 90% of the duration of action potential (APD_90_) in cardiomyocytes was not changed in the presence of acetate (*p* > 0.05, Figures 5D,E). Overall, our findings indicated that the inhibition of contraction and calcium transient in ventricular myocytes by acetate was not achieved by altering the activity of the L-type calcium channel.

### 3.6 GLPG0974 prevents acetate-induced inhibition of myocardial contraction

**FIGURE 6:**
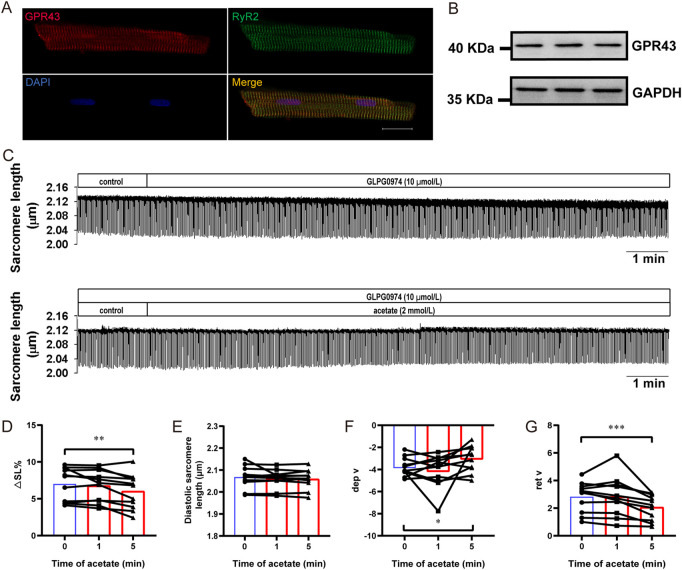
GLPG0974 prevented the acetate-induced inhibition of myocardial contraction. Photomicrographs showing GPR43 and RyR2 were co-expressed in myocardial cell **(A)**, GPR43 was labeled red, RyR2 was labeled red green and DAPI was labeled blue in the nucleus; scale bar, 20 μm. The GPR43 protein expression in myocardial cells was observed by Western blot **(B)**. Representative traces of myocardial contraction in isolated myocardial cells **(C)**. The sarcomere contraction amplitude **(D)**, diastolic sarcomere length **(E)**, maximum contraction velocity **(F)**, and maximum relaxation velocity **(G)** in the isolated myocardial cell. *N* = 5, *n* = 11. N: the number of rats, n: the number of cells. ^*^ *p* < 0.05, ^**^ *p* < 0.01, ^***^ *p* < 0.001.

We further evaluated the contribution of GPR43 to the inhibitory effect of acetate on cardiomyocyte contraction. The immunofluorescence experiment showed that GPR43 was expressed in cardiomyocytes and co-expressed with RyR2 (Figure 6A). The expression of GPR43 in the myocardial cells was demonstrated by Western blot experiment (Figure 6B). Then we measured the contractility of cardiomyocytes by using GLPG0974 to depress GPR43 activity (Figure 6C). GLPG0974 *per se* had no effect on myocardial cell contraction (Figure 6C). After GLPG0974 administration, no significant differences were found in contractile amplitude, diastolic sarcomere length, and diastolic and systolic rates between acetate treatment at 0 and 1 min (*p* > 0.05, Figures 6D–G). However, acetate inhibited the contractile amplitude and the contraction and relaxation velocity after 5 min of GLPG0974 treatment (*p* < 0.05, Figures 6D,F,G). These observations suggested that GLPG0974 could prevent acetate-induced acute inhibition of myocardial contraction.

## 4 Discussion

In the present study, acetate acutely inhibited myocardial contraction, followed by recovery in Langendorff-perfused hearts and isolated cardiomyocytes. Acetate could reduce the calcium concentration of cardiomyocytes during contraction, and such an acetate’s inhibitory effect on cardiomyocyte contraction was blocked by the GPR43 antagonist GLPG0974.

The effect of acetate on heart function has been largely understudied, and its effect on myocardial contractility remains controversial. In the present study, we found that acetate could transiently and reversibly inhibit the contraction of isolated cardiomyocytes, consistent with a previous report (Schooley et al., 2014). Acetate also transiently inhibited the LVDP in the Langendorff-perfused heart, consistent with our results tested in cardiomyocytes *in vitro* but inconsistent with Poll et al.'s study, which reported that LVDP was not impacted by acetate (Poll et al., 2021). However, we show that acetate did not significantly alter stroke volume in the whole animal experiment. It is well-established that in addition to myocardial contractility, stroke volume is affected by both preload (such as returned blood volume) and afterload (such as arterial blood pressure) (Bruss and Raja, 2022). Non-etheless, it should be borne in mind that the contractility of cardiomyocytes is also influenced by neurological components, such as sympathetic and parasympathetic control (Aksu et al., 2021). It has been reported that SCFAs regulate the autonomic nervous system and arterial blood pressure (Bruning et al., 2020; Poll et al., 2020), which supports our negative findings for changes in stroke volume induced by acetate in the whole animal experiment. In the present study, the differential effects of acetate on heart rate in both whole animals and isolated heart preparation indicated that acetate might also somehow affect autonomic control of the heart.

It has been generally accepted that membrane depolarization can activate L-type calcium channels, which in turn stimulate the sarcoplasmic reticulum ryanodine receptor and is mediated by calcium-induced calcium release, thus generating cellular calcium transients and triggering synchronous contraction of cardiomyocytes (Bers, 2002). Indeed, calcium plays a predominant role in the contraction of cardiomyocytes. In this respect, during cardiomyocyte contraction, calcium ions in the cytoplasm are derived from calcium influx through L-type calcium channels and calcium release through the sarcoplasmic reticulum (Eisner et al., 2017). In the present study, acetate significantly inhibited calcium transient, while the cardiomyocyte action potential duration and the L-type calcium current were not significantly changed. These findings suggested that acetate could inhibit calcium release from the sarcoplasmic reticulum, although further experiments are warranted to confirm this finding.

SCFAs are well-recognized to mediate various regulatory and signaling functions either by alterations in histone acetylation or as ligands activating G-protein coupled receptors, including GPR41, GPR43 and GPR109A (Kimura et al., 2020). GPR41 and GPR43 are also known as free fatty acids receptors (FFARs), FFAR3 and FFAR2, respectively. In the present study, immunohistochemistry and Western blot validated the expression of GPR43 in cardiomyocytes. GPR43 combines with Gi/o protein, inhibiting AC and decreasing intracellular cAMP levels. On the other hand, GPR43 can also couple to Gq/11 protein, increasing intracellular Ca^2+^ levels (Le Poul et al., 2003). It has been reported that acetate is more selective for GPR43 (Le Poul et al., 2003). Therefore, we speculated that acetate exerts an inhibitory effect on cardiomyocyte contraction by activating GPR43, which was validated herein by the GPR43 antagonist GLPG0974 (Namour et al., 2016).

After a temporary inhibitory effect was induced by acetate, the contraction gradually returned to normal levels in isolated hearts and cardiomyocytes, however, the calcium transient did not recover synchronously. This finding indicated that rapid recovery from contraction inhibition by acetate is independent of calcium ions. Intracellularly, acetate can be converted to acetyl-CoA by acetyl-CoA synthetases AceCS2 in cardiomyocyte mitochondria and incorporated in the tricarboxylic acid cycle and ATP production (Fujino et al., 2001; Hernandez et al., 2019). Therefore, on the one hand, acetate can enhance myocardial contraction by promoting energy metabolism. On the other hand, acetate inhibits myocardial contraction by decreasing the intracellular Ca^2+^concentration. Thus the effect of acetate on myocardial contraction depends on the balance between the two factors. This may explain the inconsistent results of previous studies, and the recovery after transient inhibition of myocardial contraction by acetate.

Free fatty acid oxidation provides the main energy source for the normal heart to sustain contractile function (Murashige et al., 2020). The failing heart is now well established as an “engine out of fuel”. The pathophysiologic transformation from normal heart to pathologic remodeling consists of fuel metabolic transitions include reduction in fatty acid oxidation, impairments in glucose metabolism, and ultimately switch to ketone body fuel utilization in heart failure (Selvaraj et al., 2020). Ketone supplementation has been shown to ameliorate heart failure in humans and mice (Horton et al., 2019; Nielsen et al., 2019). More recently, SCFA was shown more readily oxidized than ketones in failing hearts, suggesting that SCFA are a preferred energy source than ketones (Carley et al., 2021). Therefore, the acute myocardial depressant effect of SCFA needs to be considered on heart failure condition according to the present study.

## 5 Conclusion

In summary, acetate transiently inhibits contraction *via* the short-chain fatty acid receptor GPR43 in cardiomyocytes. These findings broaden our understanding of the role of short-chain fatty acids and their receptors in myocardial energy metabolism and contractile function.

## Data Availability

The raw data supporting the conclusion of this article will be made available by the authors, without undue reservation.
